# The Origin of Cancer

**Published:** 1900-03-10

**Authors:** 


### THE ORIGIN OF CANCER.

At a recent meeting of the New York Academy of Medicine Dr. Roswell Park and Dr. Gaylord presented reports upon their researches in the etiology of cancer at the State Laboratory at Buffalo, Dr. Gaylord dealing more especially with the pathological side of the question. He said that from our present knowledge it was not at all improbable that the so-called "fuchsin bodies" of Russell might after all have some definite relation to cancer. At the State Laboratory they had once succeeded in producing carcinoma by inoculation with the organism referred to. In their recent experiments they had thoroughly dried and powdered certain infected lymph nodes, which microscopic examination had showed to contain Russell's bodies, and had injected the powder into the bodies of animals. The latter had all died within three or four weeks, and not only had their lymph nodes been found generally enlarged, but these enlargements had been found to contain large numbers of Russell's bodies. This, however, is very different from the production of a cancer, and the research does not appear to have advanced matters much further.

